# Supplementary material for: Prevalence and risk of spinal pain among physiotherapists in Poland
Source: PeerJ. 2021 Jul 6;9:e11715. doi: 10.7717/peerj.11715 (PMC8269737; doi:10.7717/peerj.11715)
Supplement: Supplemental Information 3 [file peerj-09-11715-s003.docx]

**Questionaire - occurrence of spinal pain in the professional group of physiotherapists**

*Dear Ladies and Gentlemen, the following anonymous questionnaire is to draw attention to physiotherapist's occupational diseases. The questionnaire concerns the occurrence of spinal pain.*

*It consists of 3 sections.*

*(1) The first section consists of 22 questions and applies to all the Dentists.*

*(2) The second section consists of 10 questions and concerns the Professional who has experienced pain in the cervical part of the spine.*

*(3) The third section consists of 10 questions and refers to the Professional who has experienced pain in the lumbar region of the spine.*

*Thank you very much for completing the questionnaire.*

1. year of birth

2. sex:

- Woman

- Male

3. height [cm]

4. Weight [kg]

5. Professional seniority [in years]

6. What field of physiotherapy do you work in most often?

- Physical Therapy

- Kinesitherapy

- Massage

7. How many hours a day do you work with a patient?

- under 6 h

- from 6 to 8 h

- over 8 h

8. Have you experienced any back pain (both in your free time and during your work) during your work? (single choice)

- Yes

- No

9. When the first spinal pain incident occurred

- 1 year ago

- 2-3 years ago

- 4-6 years ago

- 7 - 9 years ago

- 10 or more years ago

10. number of pain episodes in the past

- 0

- 1-5

- 6-10

- over 11

11. The nature and frequency of pain

- Constant (I'm still in pain, all day and often at night of varying intensity)

- The pain comes and goes once a day

- The pain comes and goes once a week

- The pain appears and disappears once a month

- The pain appears and disappears once a year

- The pain appeared and subsided several times in my professional life

- The pain came and went only once in my professional life

12. Location of pain (several answers can be chosen)

- Cervical part of the spine - central

- Cervical part of the spine - with radiation to one limb

- Cervical part of the spine - with radiation to both limbs

- Thoracic part of the spine - centrally

- Thoracic part of the spine - with radiation

- The lumbar and sacral part of the spine - central

- Lumbar-sacral part of the spine - with radiation to one limb

- Lumbar-sacral part of the spine - with radiation to both limbs

13. intensity of the LAST Pain Episode (VAS scale)

- 0 (no pain)

- 1 (very mild)

- 2 (mild)

- 3 (mild/moderate)

- 4 (moderate)

- 5 (moderate/strong)

- 6 (strong)

- 7 (strong/very strong)

- 8 (very strong)

- 9 (very strong/stronger imaginable pain)

- 10 (strongest imaginable pain)

14. Painkillers frequency

- No painkillers

- Shortly afterwards

- Still - small doses

- Still - high doses

- Still - very high doses

15. Need for treatment (other than painkillers)

- None - I'm not healing

- None - I'm treating myself - Dentistry/osteopathy

- I'm privately receiving treatment from another Dentist/staff.

- I'm taking advantage of NFZ rehabilitation treatment

16. Restriction of physical activity

- None

- Partial

- Difficult to work

- Preventing self-functioning

17. The most common cause of spinal pain (several can be highlighted)

- Lifting

- Thorax twist

- Bow of the trunk

- Thorax overgrowth

- Push of the weight

- Pulling the weight

- Elusive

18. actions and body positions that exacerbate pain (several can be marked)

- State

- Lifting

- Bow of the trunk

- Seat

- Thorax twist

- Thorax prolapse

- Pulling the weight

19. during the last 7 days, has he/she performed any activities that require intensive physical effort (causing very fast breathing and very fast heartbeat, e.g. aerobics, fast running, fast cycling). These are activities that last at least 10 minutes.

- NO

- YES - 1 time a week

- YES - 2 times a week

- YES - 3 times a week

- YES - 4 times a week

- YES - 5 times a week

- YES - 6 times a week

- YES - 7 times a week

20. The average duration of intensive physical activity is

- except

- 10-30 minutes

- 30-50 minutes

- 50 minutes and more

21. has he or she carried out any activities in the last 7 days that require moderate, medium physical effort (causing breathing a little faster and heartbeat a little faster, e.g. cycling, playing volleyball, very fast walking)? These are activities that last at least 10 minutes.

- NO

- YES- once a week

- YES- 2 times a week

- YES- 3 times a week

- YES- 4 times a week

- YES- 5 times a week

- YES- 6 times a week

- YES- 7 times a week

22. the average duration of moderate, average physical effort is

- except

- 10-30 minutes

- 30-50 minutes

- 50 minutes and more

**NDI (Neck Disability Index) Cervical Disability Index**

Please fill in if you have pain in the cervical part of your spine

(Please note that there is a 3rd section on lumbar pain in the spine)

1. Pain severity

- I'm not feeling any pain right now.

- The pain is mild at the moment.

- The pain appears and goes away and is moderate.

- The pain is moderate and basically constant.

- The pain is strong, it goes away and it comes.

- The pain is strong, it goes away and it comes. The pain is strong and does not change much.

2. Daily activities (washing, dressing, etc.)

- I can do my own daily activities without any additional pain.

- I can do my own daily activities, but they cause considerable pain.

- It is very painful to do everyday activities on my own, I do them slowly and carefully.

- I need some help with my daily activities, but most of the activities are on my own...

- I need help every day to do most things.

- I can't get dressed, I wash myself with difficulty, I spend most of my time in bed.

3. Pick up

- I can lift heavy objects without any additional pain.

- I can lift heavy objects, but it causes extra pain.

- I can't lift heavy objects from the floor because of the pain, but I can lift them when it is convenient...

- Because of the pain, I can't lift heavy objects, but I can lift light or medium-heavy, ...

- Because of the pain, I can only lift very light objects.

- I can't pick up any objects because of the pain.

4. Reading

- I can read as much as I want without feeling any pain in the neck.

- I can read as much as I want, but reading causes a small, additional pain in the neck.

- I can read as much as I want, but reading causes a slight additional neck pain of medium intensity.

- I have to limit my reading because of the extra pain of medium intensity.

- I can hardly read at all because of the severe pain.

- I can't read at all because of the neck pain.

5. Headache

- I don't have a headache.

- Headaches are rare and minor.

- The headache is rare and medium.

- I often have moderate headaches.

- I often have severe headaches.

- I have headaches almost all the time.

6. Concentration

- I can always concentrate fully without difficulty.

- I can always concentrate fully, though with a little difficulty.

- I have some difficulty concentrating.

- I have considerable difficulty concentrating

- I'm having a lot of trouble concentrating.

- I can't concentrate at all.

7. The job

- I can work without limitations.

- I can only do routine work, but no more.

- I can do most of my mandatory work activities.

- I am not able to carry out routine work activities.

- I can do any kind of work with great difficulty.

- I am not able to work because of the pain.

8. Driving

- I can drive a car and it doesn't cause additional neck pain.

- I can drive without any restrictions, but this is accompanied by a slight additional pain in the neck.

- I can drive without restrictions, but it is accompanied by a slight additional neck pain of medium intensity...

- I can't drive as much as I want to because of a medium neck pain.

- I hardly ever drive at all because of severe neck pain.

- I can't drive at all because of neck pain.

9. Sleeping

- Pain doesn't disturb my sleep at all.

- Pain disturbs my sleep very little (less than 1 hour of insomnia per day).

- Pain disturbs my sleep to a small extent (1-2 hours of insomnia per day).

- The pain interferes with my sleep to a medium degree (2-3 hours of insomnia per day).

- Pain disturbs my sleep significantly (3-5 hours of insomnia per day).

- The pain prevents me from sleeping at all (5-7 hours of insomnia per day).

10. Recreation

- I can do all my recreational activities without pain.

- I can do all my recreational activities with a little neck pain.

- I can do most, but not all recreational activities because of neck pain.

- I am not able to do most typical recreational activities because of neck pain.

- I can hardly do any recreational activities due to neck pain.

- I do not do recreational activities at all because of neck pain.

**ODI (Oswestry Disability Index) malfunction indicator**

**Please fill in if you have pain in the lumbar part of your spine**

1. Pain severity

- I'm not feeling any pain right now.

- At the moment, the pain is small.

- The pain is medium at the moment.

- The pain is quite severe at the moment.

- The pain is very strong at the moment.

- At the moment the pain is the worst I can imagine.

2. Daily activities (washing, dressing, etc.)

- I can do my own daily activities without any additional pain.

- I can do my own daily activities, but they cause considerable pain.

- It is very painful to do everyday activities on my own, I do them slowly and carefully.

- I need some help with my daily activities, but most of them are independent...

- I need help every day to do most things.

- I can't get dressed, I wash myself with difficulty, I spend most of my time in bed.

3. Walking

- The pain doesn't stop me from walking.

- Pain doesn't allow me to walk more than 1 - 2 km.

- The pain doesn't let me walk more than 500 m.

- Pain doesn't let me walk more than 100 m.

- I can only walk with a stick or crutches.

- I stay in bed all the time, I find it very difficult to get to the toilet.

4. lifting

- I can lift heavy objects without any additional pain.

- I can lift heavy objects, but it causes extra pain.

- Because of the pain, I cannot lift heavy objects from the floor, but I can lift them when they are conveniently placed, for example on a table.

- Because of the pain, I cannot lift heavy objects, but I can lift light or medium heavy when conveniently placed, for example on a table.

- Because of the pain, I can only lift very light objects.

- Because of the pain, I cannot lift or carry any objects.

5. Seat

- I can sit in any chair any amount of time.

- I can sit in my favorite chair any amount of time.

- I can sit for no more than 1 hour because of the pain.

- Because of the pain, I can sit no more than half an hour.

- I can sit for no more than 10 minutes because of the pain.

- I can't sit at all because of the pain.

6. State

- I can stand any amount of time without any additional pain.

- I can stand any amount of time, but it causes extra pain.

- Because of the pain, I cannot stand for more than 1 hour.

- I can't stand more than 30 minutes because of the pain.

- I can't stand more than 10 minutes because of the pain.

- I can't stand at all because of the pain.

7. Sleeping

- My sleep is never disturbed by pain.

- My sleep is sometimes disturbed by pain.

- I don't sleep more than 6 hours because of the pain.

- I don't sleep for more than 4 hours because of the pain.

- I do not sleep for more than 2 hours because of the pain.

- I can't sleep at all because of the pain.

8. Sexual life (if applicable)

- My sex life looks the same as usual and does not cause any additional pain.

- My sex life looks the same as usual, but causes extra pain.

- My sex life looks roughly the same as usual, but causes severe pain.

- My sex life is severely limited by pain.

- My sex life is almost nonexistent because of the pain.

- I can't have sex at all because of the pain.

9. Social life

- My social life is normal, no additional pain.

- My social life is normal, but it causes extra pain.

- Pain has no significant impact on my social life, except for activities related to physical activity (e.g. sport).

- The pain has limited my social life, so I stay home more often.

- Pain has limited my social life to meetings at home.

- Because of the pain, I don't have a social life.

10. Travelling

- I can travel anywhere without pain.

- I can travel anywhere, but it causes extra pain.

- The pain is strong, but I can travel more than 2 hours.

- The pain does not allow me to travel more than 1 hour.

- The pain limits my travel to necessary travels of less than 30 minutes.

- Because of the pain I travel only to places related to the treatment.
