# Supplementary material for: Prevalence and risk of spinal pain among physiotherapists in Poland
Source: PeerJ. 2021 Jul 6;9:e11715. doi: 10.7717/peerj.11715 (PMC8269737; doi:10.7717/peerj.11715)
Supplement: Supplemental Information 2 [file peerj-09-11715-s002.pdf]

Ankieta

Pytania | Odpowiedzi 242

Sekcja 1 z 3

Ankieta - występowanie dolegliwości bólowych kręgosłupa w grupie zawodowej fizjoterapeutów

Szanowni Państwo, poniższa anonimowa ankieta ma na celu zwrócenie uwagi na choroby zawodowe Fizjoterapeuty.
Ankieta dotyczy występowania dolegliwości bólowych kręgosłupa.
Składa się z 3 sekcji.
(1) Pierwsza sekcja składa się z 22 pytań i dotyczy wszystkich fizjoterapeutów.
(2) Druga sekcja składa się z 10 pytań i dotyczy fizjoterapeutów którzy doświadczyli dolegliwości bólowych w części szyjnej kręgosłupa.
(3) Trzecia sekcja składa się z 10 pytań i dotyczy fizjoterapeutów którzy doświadczyli dolegliwości bólowych w części lędźwiowej kręgosłupa.
Serdecznie dziękujemy za wypełnienie ankiety.

1. Rok urodzenia *

Tekst krótkiej odpowiedzi

2. Płeć *

- Kobieta
- Mężczyzna

3. Wzrost [cm] *

Tekst krótkiej odpowiedzi

4. Waga [kg] *

Tekst krótkiej odpowiedzi

5. Staż zawodowy [w latach] *

Tekst krótkiej odpowiedzi

6. W jakiej dziedzinie fizjoterapii pracuje Pani/Pan najczęściej? *

- Fizykoterapia
- Kinezyterapia
- Masaż

7. Ile godzin dziennie pracuje Pani/Pan z pacjentem? *

- poniżej 6 h
- od 6 do 8 h
- powyżej 8 h

8. Czy wystąpiły u Pani/Pana w okresie wykonywania pracy zawodowej dolegliwości bólowe kręgosłupa (zarówno

Jednokrotny wybór

- Tak
- Nie
- Dodaj opcję lub dodaj opcję „Inne"

Wymagane

9. Kiedy wystąpił pierwszy incydent bólu kręgosłupa

- 1 rok temu
- 2-3 lata temu
- 4-6 lat temu
- 7 - 9 lat temu
- 10 i więcej lat temu

10. Liczba epizodów bólowych w przeszłości

- 0
- 1-5
- 6-10
- powyżej 11

11. Charakter bólu i jego częstotliwość

- Stały (ciągle towarzyszy mi ból, cały dzień i często w nocy o zmiennym nasileniu)
- Ból pojawia się i ustępuje raz na dzień
- Ból pojawia się i ustępuje raz w tygodniu
- Ból pojawia się i ustępuje raz w miesiąc
- Ból pojawia się i ustępuje raz w roku
- Ból pojawił się i ustąpił kilka razy w moim życiu zawodowym
- Ból pojawił się i ustąpił tylko raz w moim życiu zawodowym

12. Lokalizacja bólu (można wybrać kilka odpowiedzi)

- Część szyjna kręgosłupa - centralnie
- Część szyjna kręgosłupa - z promieniowaniem do jednej kończyny
- Część szyjna kręgosłupa - z promieniowaniem do obu kończyn
- Część piersiowa kręgosłupa - centralnie
- Część piersiowa kręgosłupa - z promieniowaniem
- Część lędźwiowo-krzyżowa kręgosłupa - centralnie
- Część lędźwiowo-krzyżowa kręgosłupa - z promieniowaniem do jednej kończyny
- Część lędźwiowo-krzyżowa kręgosłupa - z promieniowaniem do obu kończyn
- Inna odpowiedź...

13. Intensywność OSTATNIEGO epizodu bólu (skala VAS)

- 0 (brak bólu)
- 1 (bardzo łagodny)
- 2 (łagodny)
- 3 (łagodny/umiarkowany)
- 4 (umiarkowany)
- 5 (umiarkowany/silny)
- 6 (silny)
- 7 (silny/bardzo silny)
- 8 (bardzo silny)
- 9 (bardzo silny/najsilniejszy wyobrażalny ból)
- 10 (najsilniejszy wyobrażalny ból)

14. Częstotliwość zażywania środków przeciwbólowych

- Brak środków przeciwbólowych
- Doraźnie
- Ciągle – małe dawki
- Ciągle – duże dawki
- Ciągle – bardzo duże dawki

15. Konieczność korzystania z leczenia (innego niż leki przeciwbólowe)

- Brak – nie leczę się
- Brak – leczę się sam – fizjoterapia/osteopatia
- Korzystam prywatnie z leczenia u innego fizjoterapeuty/osteopaty
- Korzystam z leczenia rehabilitacyjnego NFZ

16. Ograniczenie aktywności ruchowej

- Żadne
- Częściowe
- Utrudniające pracę
- Uniemożliwiające samodzielne funkcjonowanie

17. Najczęstsza przyczyna występowania dolegliwości bólowych kręgosłupa (można zaznaczyć kilka)

- Dźwiganie
- Skręt tułowia
- Skłon tułowia
- Przeprost tułowia
- Pchnięcie ciężaru
- Pociągnięcie ciężaru
- Nieuchwytna

18. Czynności i pozycje ciała nasilające ból (można zaznaczyć kilka)

- Stanie
- Dźwiganie
- Skłon tułowia
- Siedzenie
- Skręt tułowia
- Przeprost tułowia
- Pociągnięcie ciężaru

19. Czy w ciągu ostatnich 7 dni wykonywał/a P. czynności wymagające intensywnego wysiłku fizycznego (powodującego bardzo szybkie oddychanie i bardzo szybkie bicie serca, np. aerobik, szybki bieg, szybka jazda rowerem). Chodzi o czynności trwające minimum 10 minut. *

- NIE
- TAK - 1 razy w tygodniu
- TAK - 2 razy w tygodniu
- TAK - 3 razy w tygodniu
- TAK - 4 razy w tygodniu
- TAK - 5 razy w tygodniu
- TAK - 6 razy w tygodniu
- TAK - 7 razy w tygodniu

20. Przeciętny czas trwania intensywnego wysiłku fizycznego to *

- nie dotyczy
- 10-30 minut
- 30-50 minut
- 50 minut i więcej

21. Czy w ciągu ostatnich 7 dni wykonywał/a P. czynności wymagające umiarkowanego, średniego wysiłku fizycznego (powodującego trochę szybsze oddychanie i trochę szybsze bicie serca, np. jazda rowerem, gra w siatkówkę, bardzo szybki marsz). Chodzi o czynności trwające minimum 10 minut. *

- NIE
- TAK- 1 raz w tygodniu
- TAK- 2 razy w tygodniu
- TAK- 3 razy w tygodniu
- TAK- 4 razy w tygodniu
- TAK- 5 razy w tygodniu
- TAK- 6 razy w tygodniu
- TAK- 7 razy w tygodniu

22. Przeciętny czas trwania umiarkowanego, średniego wysiłku fizycznego to *

- nie dotyczy
- 10-30 minut
- 30-50 minut
- 50 minut i więcej

Po sekcji 1 Przejdź do następnej sekcji

Sekcja 2 z 3

NDI (Neck Disability Index) Wskaźnik niepełnosprawności szyjnej

Proszę o wypełnienie jeżeli ma Pani/Pan dolegliwości bólowe w części szyjnej kręgosłupa

(Proszę pamiętać, że istnieje jeszcze 3 sekcja dotycząca dolegliwości bólowych w części lędźwiowej kręgosłupa)

1. Nasilenie bólu

- W tej chwili nie odczuwam bólu.
- W tej chwili ból jest łagodny.
- Ból pojawia się i odchodzi i jest umiarkowany.
- Ból jest umiarkowany i w zasadzie stały.
- Ból jest silny, odchodzi i przychodzi.
- Ból jest silny, odchodzi i przychodzi. Ból jest silny i nie bardzo się zmienia.

2. Codzienne czynności (mycie, ubieranie, itp.)

- Mogę wykonywać samodzielnie codzienne czynności bez dodatkowego bólu.
- Mogę wykonywać samodzielnie codzienne czynności, ale wywołują one znaczny ból.
- Samodzielne wykonywanie codziennych czynności jest bardzo bolesne, wykonuję je powoli i uważnie.
- Wymagam pewnej pomocy przy wykonywaniu codziennych czynności, lecz większość wykonuję samodzi...
- Potrzebuję pomocy każdego dnia przy wykonywaniu większości czynności.
- Nie jestem w stanie się ubrać, myję się z trudem, większość czasu spędzam w łóżku.

3. Podnoszenie

- Mogę podnosić ciężkie przedmioty bez dodatkowego bólu.
- Mogę podnosić ciężkie przedmioty, lecz powoduje to dodatkowy ból.
- Z powodu bólu nie mogę podnosić ciężkich przedmiotów z podłogi, lecz mogę je podnosić, gdy są dogod...
- Z powodu bólu nie mogę podnosić ciężkich przedmiotów, lecz mogę podnosić lekkie lub średnio ciężkie, ...
- Z powodu bólu mogę podnosić tylko bardzo lekkie przedmioty.
- Z powodu bólu nie mogę podnosić żadnych przedmiotów.

4. Czytanie

- Mogę czytać tyle, ile chcę nie odczuwając żadnego bólu karku.
- Mogę czytać tyle, ile chcę, lecz czytanie powoduje niewielki, dodatkowy ból karku.
- Mogę czytać tyle, ile chcę, lecz czytanie powoduje dodatkowy ból karku o średnim nasileniu.
- Muszę ograniczać czytanie z powodu dodatkowego bólu o średnim nasileniu.
- Prawie wcale nie mogę czytać z powodu silnego bólu.
- Z powodu bólu karku wcale nie jestem w stanie czytać.

5. Ból głowy

- Nie miewam bólów głowy.
- Ból głowy zdarza się rzadko i jest niewielki.
- Ból głowy zdarza się rzadko i ma średnie nasilenie.
- Często miewam bóle głowy o średnim nasileniu.
- Często miewam silne bóle głowy.
- Głowa boli mnie prawie cały czas.

6. Koncentracja

- Mogę się zawsze w pełni skoncentrować bez trudu.
- Mogę się zawsze w pełni skoncentrować, choć z niewielkimi trudnościami.
- Mam pewne trudności z koncentracją.
- Mam znaczne trudności z koncentracją
- Mam bardzo duże trudności z koncentracją.
- W ogóle nie mogę się skoncentrować.

7. Praca

- Mogę pracować bez ograniczeń.
- Mogę wykonywać wyłącznie rutynowe czynności w pracy, lecz nie więcej.
- Mogę wykonywać większość moich obowiązkowych czynności w pracy.
- Nie jestem w stanie wykonywać rutynowych czynności w pracy.
- Z wielkim trudem wykonuję jakąkolwiek pracę.
- Z powodu bólu nie jestem w stanie pracować.

8. Jazda samochodem

- Mogę jeździć samochodem i nie powoduje to dodatkowego bólu karku.
- Mogę jeździć samochodem bez ograniczeń, lecz towarzyszy temu niewielki dodatkowy ból karku.
- Mogę jeździć samochodem bez ograniczeń, lecz towarzyszy temu dodatkowy bólu karku o średnim nasile...
- Nie mogę jeździć samochodem tyle, ile chcę z powodu bólu karku o średnim nasileniu.
- Prawie wcale nie jeżdżę samochodem z powodu silnego bólu karku.
- Wcale nie mogę jeździć samochodem z powodu bólu karku.

9. Spanie

- Ból w ogóle nie zaburza mojego snu.
- Ból zaburza mój sen w bardzo niewielkim stopniu (mniej niż 1 godzina bezsenności na dobę).
- Ból zaburza mój sen w niewielkim stopniu (1-2 godziny bezsenności na dobę).
- Ból zaburza mój sen w średnim stopniu (2-3 godziny bezsenności na dobę).
- Ból zaburza mój sen w znacznym stopniu (3-5 godzin bezsenności na dobę).
- Ból w ogóle nie pozwala mi spać (5-7 godzin bezsenności na dobę).

10. Rekreacja

- Mogę wykonywać wszystkie czynności rekreacyjne bez bólu.
- Mogę wykonywać wszystkie czynności rekreacyjne z niewielkim bólem karku.
- Z powodu bólu karku mogę wykonywać większość czynności rekreacyjnych – lecz nie wszystkie.
- Z powodu bólu karku nie jestem w stanie wykonywać większości typowych czynności rekreacyjnych.
- Mogę z trudem wykonywać tylko niektóre czynności rekreacyjne z powodu bólu karku.
- Z powodu bólu karku wcale nie wykonuję czynności rekreacyjnych.

Po sekcji 2 Przejdź do następnej sekcji

Sekcja 3 z 3

ODI (Oswestry Disability Index) wskaźnik niesprawności 2.1

Proszę o wypełnienie jeżeli ma Pani/Pan dolegliwości bólowe w części lędźwiowej kręgosłupa

1. Nasilenie bólu

- W tej chwili nie odczuwam bólu.
- W tej chwili ból jest niewielki.
- W tej chwili ból jest średni.
- W tej chwili ból jest dość silny.
- W tej chwili ból jest bardzo silny.
- W tej chwili ból jest najgorszy, jaki mogę sobie wyobrazić.

2. Codzienne czynności (mycie, ubieranie, itp.)

- Mogę wykonywać samodzielnie codzienne czynności bez dodatkowego bólu.
- Mogę wykonywać samodzielnie codzienne czynności, ale wywołują one znaczny ból.
- Samodzielne wykonywanie codziennych czynności jest bardzo bolesne, wykonuję je powoli i uważnie.
- Wymagam pewnej pomocy przy wykonywaniu codziennych czynności, ale większość wykonuję samodziel...
- Potrzebuję pomocy każdego dnia przy wykonywaniu większości czynności.
- Nie jestem w stanie się ubrać, myję się z trudem, większość czasu spędzam w łóżku.

3. Chodzenie

- Ból wcale nie przeszkadza mi w chodzeniu.
- Ból nie pozwala mi chodzić dalej niż 1 – 2 km.
- Ból nie pozwala mi chodzić dalej niż 500 m.
- Ból nie pozwala mi chodzić dalej niż 100 m.
- Mogę chodzić jedynie o lasce lub o kulach.
- Pozostaję cały czas w łóżku, z wielkim trudem dostaję się do toalety.

4. Podnoszenie

- Mogę podnosić ciężkie przedmioty, bez dodatkowego bólu.
- Mogę podnosić ciężkie przedmioty, lecz powoduje to dodatkowy ból.
- Z powodu bólu nie mogę podnosić ciężkich przedmiotów z podłogi, lecz mogę podnosić je, gdy są dogod...
- Z powodu bólu nie mogę podnosić ciężkich przedmiotów, lecz mogę podnosić lekkie lub średnio ciężkie, ...
- Z powodu bólu mogę podnosić tylko bardzo lekkie przedmioty.
- Z powodu bólu nie mogę podnosić ani przenosić żadnych przedmiotów.

5. Siedzenie

- Mogę siedzieć na każdym krześle dowolną ilość czasu.
- Mogę siedzieć na moim ulubionym krześle dowolną ilość czasu.
- Z powodu bólu mogę siedzieć nie dłużej niż 1 godzinę.
- Z powodu bólu mogę siedzieć nie dłużej niż pół godziny.
- Z powodu bólu mogę siedzieć nie dłużej niż 10 minut.
- Z powodu bólu wcale nie mogę siedzieć.

6. Stanie

- Mogę stać dowolną ilość czasu bez dodatkowego bólu.
- Mogę stać dowolną ilość czasu, lecz powoduje to dodatkowy ból.
- Z powodu bólu nie mogę stać dłużej niż 1 godzinę.
- Z powodu bólu nie mogę stać dłużej niż 30 minut.
- Z powodu bólu nie mogę stać dłużej niż 10 minut.
- Z powodu bólu nie mogę w ogóle stać.

7. Spanie

- Mój sen nigdy nie jest zaburzony bólem.
- Mój sen czasem jest zaburzony bólem.
- Z powodu bólu nie śpię dłużej niż 6 godzin.
- Z powodu bólu nie śpię dłużej niż 4 godziny.
- Z powodu bólu nie śpię dłużej niż 2 godziny.
- Z powodu bólu w ogóle nie mogę spać.

8. Życie seksualne (jeśli dotyczy)

- Moje życie seksualne wygląda tak jak zwykle i nie wywołuje dodatkowego bólu.
- Moje życie seksualne wygląda tak jak zwykle, ale wywołuje dodatkowy ból.
- Moje życie seksualne wygląda w przybliżeniu tak jak zwykle, ale powoduje silny ból.
- Moje życie seksualne jest mocno ograniczone przez ból.
- Moje życie seksualne prawie nie istnieje z powodu bólu.
- Z powodu bólu w ogóle nie mogę prowadzić życia seksualnego.

9. Życie towarzyskie

- Moje życie towarzyskie jest normalne, nie powoduje dodatkowego bólu.
- Moje życie towarzyskie jest normalne, lecz powoduje dodatkowy ból.
- Ból nie ma znaczącego wpływu na moje życie towarzyskie, poza czynnościami związanymi z aktywnością...
- Ból ograniczył moje życie towarzyskie, z tego powodu częściej zostaję w domu.
- Ból ograniczył moje życie towarzyskie do spotkań w domu.
- Z powodu bólu nie prowadzę życia towarzyskiego.

10. Podróżowanie

- Mogę podróżować wszędzie bez bólu.
- Mogę podróżować wszędzie, lecz powoduje to dodatkowy ból.
- Ból jest silny, ale jestem w stanie podróżować ponad 2 godziny.
- Ból nie pozwala mi podróżować dłużej niż 1 godzinę.
- Ból ogranicza moje podróże do niezbędnych, trwających krócej niż 30 minut.
- Z powodu bólu podróżuję wyłącznie do miejsc związanych z leczeniem.
